# The Microstructure of Fe-Based Laminated Metal Composite Produced by Powder Metallurgy

**DOI:** 10.3390/ma14216533

**Published:** 2021-10-29

**Authors:** Guo-Jiun Shu, Cun-Jheng Huang, Wei-Xiang Chien, Pei Wang, Ming-Wei Wu

**Affiliations:** 1 Department of Materials and Mineral Resources Engineering, National Taipei University of Technology, Taipei 10608, Taiwan; gjshu@ntut.edu.tw (G.-J.S.); ggc555378@gmail.com (C.-J.H.); genius202026@gmail.com (W.-X.C.); 2 Additive Manufacturing Institute, Shenzhen University, Shenzhen 518060, China

**Keywords:** powder metallurgy, steel, microstructure, diffusion

## Abstract

Powder metallurgy (PM) is a versatile process to manufacture nearly net-shaped metallic materials in industry. In this study, the PM process was used to fabricate two Fe-based laminated metal composites (LMCs), Fe-4Ni-3Cr-0.5Mo-0.5C/Fe and 410/304L. The results showed that after sintering, the LMCs were free of interfacial cracks and distortion, indicating that the PM process is a feasible means for producing these LMCs. In the Fe-4Ni-3Cr-0.5Mo-0.5C/Fe LMC, the diffusion of C resulted in the generation of a continuous pearlite layer between the Fe-4Ni-3Cr-0.5Mo-0.5C and Fe layers and a ferrite/pearlite mixture in the Fe layer. In the 410/304L LMC, the difference in the chemical potentials of C between the 304L and 410 layers led to the uphill diffusion of C from the 410 layer to the 304L layer. A continuous ferrite layer was thus formed near the interface of the 410 layer. Furthermore, a martensite layer of about 50 μm thickness was generated at the interface due to the high Cr and Ni content.

## 1. Introduction

Alloying elements or heat treatment can be used to increase the strength and hardness of metallic materials, but at the cost of ductility and toughness. This strength–ductility tradeoff is the greatest challenge in the design of structural metallic materials. Composite materials in various forms have been widely studied due to their versatility [1,2,3,4,5]. To obtain a metallic material with both high strength and high ductility, numerous studies have been conducted to produce laminated metal composites (LMCs) by combining one metal with high strength and another metal with high ductility [6,7]. 

In general, the major technique for fabricating LMCs is rolling to bond the metal sheets, and heat treatment is applied to facilitate the diffusion and bonding between the neighboring metal sheets [6,7,8,9]. Huang et al. [10] produced a Ti/Al LMC with a thickness of about 100 μm per layer by hot rolling and found that this LMC exhibited outstanding mechanical properties. The yield strengths of pure Ti and pure Al are 331 MPa and 25 MPa, respectively. The yield strength of the Ti/Al LMC is 175 MPa, which conforms to the rule of mixture. The elongations of pure Ti and pure Al are, respectively, 13% and 24%. However, the elongation of the Ti/Al LMC is as high as 43%, nearly double that of pure Al. These findings indicate that LMCs are promising structural materials. 

Powder metallurgy (PM) is a cost-effective process for manufacturing nearly net-shaped metallic materials, particularly Fe-based alloys, in mass production, one that has been widely investigated [11,12,13,14,15]. However, the use of PM technology to fabricate LMCs has been rare to date in industry. In the PM field, the Fe-based alloys, including alloy and stainless steels, comprise 70% of the output value. The aim of this study was thus to produce alloy steel (Fe-4Ni-3Cr-0.5Mo-0.5C/Fe) and stainless steel (410/304L) LMCs by PM process, wherein the interfacial bonding, microstructure, and elemental distributions were analyzed to understand the feasibility of the PM process for LMCs. For the alloy steel LMC, Fe-4Ni-3Cr-0.5Mo-0.5C steel was chosen as the high-strength layer due to its superior combination of strength and toughness [16]. Pure Fe was selected as the high-ductility layer due to its high ductility, although the strength of pure Fe is low. On the other hand, in the stainless steel LMC, the 410 martensitic and 304L austenitic stainless steel layers were, respectively, the high-strength and high-ductility layers. 

## 2. Experimental Procedure

Alloy steel (Fe-4Ni-3Cr-0.5Mo-0.5C/Fe) and stainless steel (410/304L) LMCs were fabricated and investigated in this study. For the Fe-4Ni-3Cr-0.5Mo-0.5C/Fe LMC, the Fe-4Ni-3Cr-0.5Mo-0.5C and pure Fe layers served as the high-strength and high-ductility layers, respectively. The Fe-4Ni-3Cr-0.5Mo-0.5C layer was made with Fe-3Cr-0.5Mo pre-alloyed powder (Höganäs AB, Höganäs, Sweden), 4 wt % pure nickel powder (Vale, Sudbury, Canada), and 0.5 wt % graphite powder (Asbury Carbons, Asbury, NJ, USA), and the pure Fe layer was prepared with pure iron powder (Höganäs AB, Höganäs, Sweden). For the 410/304L LMC, the 410 and 304L layers were, respectively, the high-strength and high-ductility layers. The 410 layer was produced by mixing 410L (Fe-12.5Cr, Daido Steel Co., Ltd., Nagoya, Japan) and 0.4 wt % graphite powder. Decarburization occurred during sintering, and therefore 0.4 wt % graphite powder was added to the 410L powder to obtain 100% martensite after sintering. The 304L (Fe-18.5Cr-11.3Ni, Daido Steel Co., Ltd., Nagoya, Japan) powder was used to produce the 304L layer. To increase the powder compressibility, we added 0.75 wt % Acrawax to the previous powder mixtures as a lubricant, and the mixtures were blended in a V-cone mixer for 1 h. 

To increase the ductility of the high-strength steel without obvious sacrifice to the strength, we fixed the volume percentages of the high strength and high ductility layers at 80:20, and three-layer green specimens with a thickness ratio of 2:1:2 (high-strength layer/high-ductility layer/high-strength layer) were produced. By controlling the thicknesses of the three-layer green parts at a ratio of 2:1:2, we were able to obtain the LMCs with a ratio of 80 vol % high-strength layer and 20 vol % high-ductility layer. After each layer of powder was added, they were pre-pressed at 20 MPa to ensure the flatness of the powder in each layer. After three layers of the powders were added to the mold in order, the powders were uniaxially compacted at 700 MPa to prepare disc-shaped green specimens with a diameter of 13 mm and a height of 4.4 mm. For debinding, the green specimens were heated at 5 °C/min to 550 °C and then held at that temperature for 15 min in 91N_2_:9H_2_. The debound Fe-4Ni-3Cr-0.5Mo-0.5C/Fe specimens were heated at 10 °C/min to 1250 °C and held at that temperature for one hour in 91N_2_:9H_2_, followed by furnace cooling. For the 410/304L LMC, the debound specimens were heated at 10 °C/min to 1120 °C and held at that temperature for one hour in a vacuum furnace. The monolithic Fe-4Ni-3Cr-0.5Mo-0.5C, Fe, 410, and 304L sintered specimens were also prepared by the above procedures for comparison. 

The sintered density was measured by Archimedes’ method as per MPIF (Metal Powder Industrial Federation) standard 42. The metallographic specimens were prepared and etched with 100 mL alcohol + 2 mL nitric acid + 4 g picric acid for the Fe-4Ni-3Cr-0.5Mo-0.5C/Fe and aqua regia for the 410/304L. An optical microscope and a scanning electron microscope (SEM, JSM-6510LV, JEOL, Tokyo, Japan) were used to observe the microstructure near the interface. The elemental distributions after sintering were identified by electron probe micro-analysis (EPMA, JXA-8200SX, JEOL, Tokyo, Japan). The crystal structure near the interface was analyzed by electron backscatter diffraction (EBSD, NordlysNano, Oxford, UK). The microhardness of various microstructures was determined using a Vickers microhardness tester (MMX-T, MATSUZAWA, Tokyo, Japan) with a loading of 5 gf due to the small size of a specific area.

## 3. Results and Discussion

### 3.1. Density

**Figure 1:**
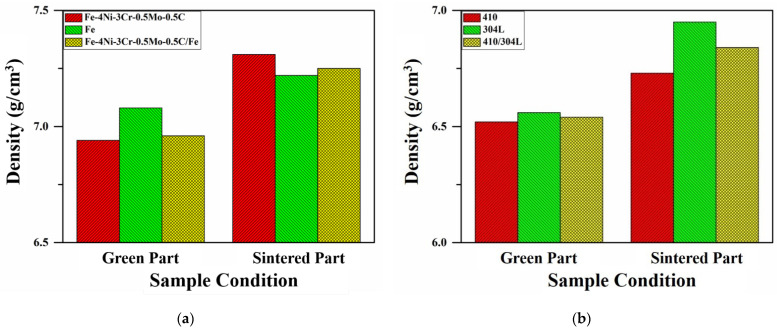
The green and sintered densities of the LMCs and the monolithic specimens. (**a**) Fe-4Ni-3Cr-0.5Mo-0.5C/Fe; (**b**) 410/304L.

The green and sintered densities of the LMCs and the monolithic specimens were examined and are shown in Figure 1. The green densities of the Fe-4Ni-3Cr-0.5Mo-0.5C/Fe LMC, the monolithic Fe-4Ni-3Cr-0.5Mo-0.5C, and the monolithic Fe specimens were 6.96 g/cm^3^, 6.94 g/cm^3^, and 7.08 g/cm^3^, respectively. After sintering, the sintered densities of the Fe-4Ni-3Cr-0.5Mo-0.5C/Fe LMC, the monolithic Fe-4Ni-3Cr-0.5Mo-0.5C, and the monolithic Fe specimens were, respectively, 7.25 g/cm^3^, 7.31 g/cm^3^, and 7.22 g/cm^3^. As displayed in Figure 1, both the green and sintered densities of the Fe-4Ni-3Cr-0.5Mo-0.5C/Fe and 410/304L LMCs fell within those of the monolithic materials.

### 3.2. Microstructure of Alloy Steel LMC

**Figure 2:**
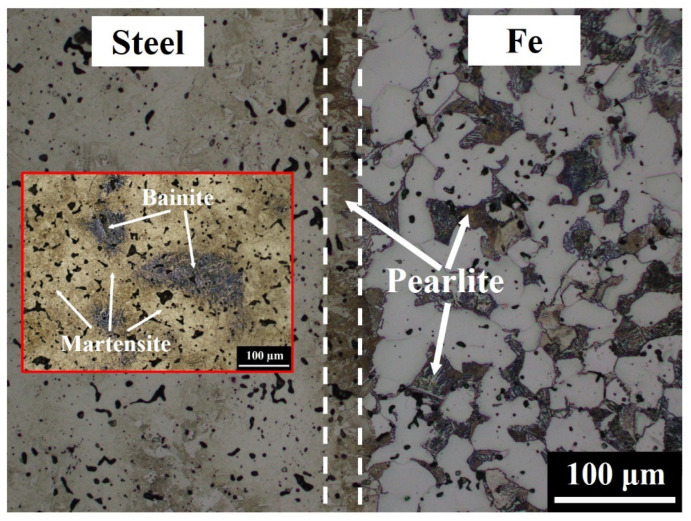
Microstructure at the interface of the Fe-4Ni-3Cr-0.5Mo-0.5C/Fe LMC.

**Figure 3:**
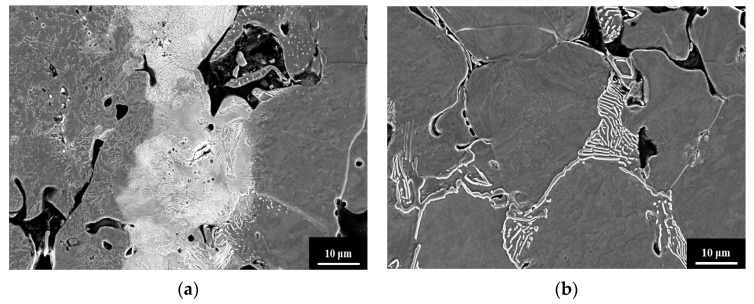
(**a**) The continuous pearlite generated at the Fe-4Ni-3Cr-0.5Mo-0.5C/Fe interface and (**b**) the pearlite formed in the pure Fe layer.

Figure 2 shows the microstructure at the interface of Fe-4Ni-3Cr-0.5Mo-0.5C/Fe LMC. The pure Fe layer should have consisted of 100% ferrite, but pearlite was also observed, as shown in Figure 2 and Figure 3. Moreover, a continuous pearlite layer with a thickness of about 30 μm existed at the interface between the Fe-4Ni-3Cr-0.5Mo-0.5C and Fe layers. The microstructures of the Fe-4Ni-3Cr-0.5Mo-0.5C layer were martensite and bainite [16,17]. In Figure 2, the Fe-4Ni-3Cr-0.5Mo-0.5C side was only slightly etched, and thus the microstructures were not very clear. If the Fe-4Ni-3Cr-0.5Mo-0.5C side were adequately etched, the continuous pearlite layer at the interface would be over-etched and relatively unobservable. For clear observation of the martensite and bainite in the Fe-4Ni-3Cr-0.5Mo-0.5C layer, a small image of the Fe-4Ni-3Cr-0.5Mo-0.5C layer adequately etched is also included in Figure 2. 

**Figure 4:**
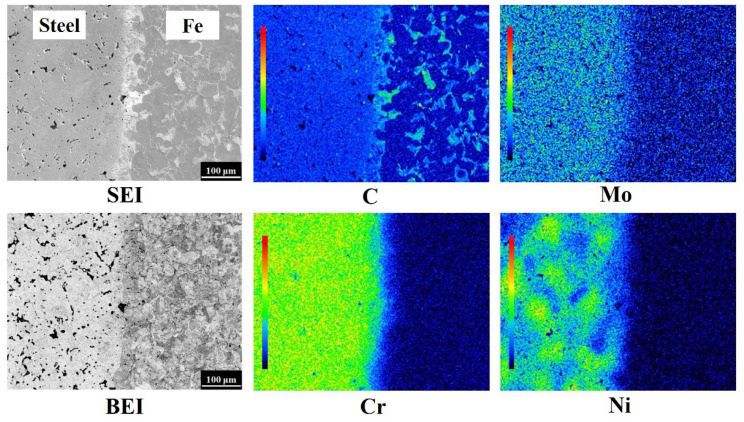
EPMA elemental distributions at the interface of the Fe-4Ni-3Cr-0.5Mo-0.5C/Fe LMC.

The elemental distributions of the Fe-4Ni-3Cr-0.5Mo-0.5C/Fe LMC were analyzed by EPMA and are shown in Figure 4. Cr, Mo, and C were homogeneously distributed in the Fe-4Ni-3Cr-0.5Mo-0.5C layer, but Ni was not uniformly distributed because elemental Ni powder was used [17]. Furthermore, after 1250 °C sintering, the C in the Fe-4Ni-3Cr-0.5Mo-0.5C layer had obviously diffused into the Fe layer, resulting in the formation of pearlite in the Fe layer. 

### 3.3. Microstructure of Stainless Steel LMC

**Figure 5:**
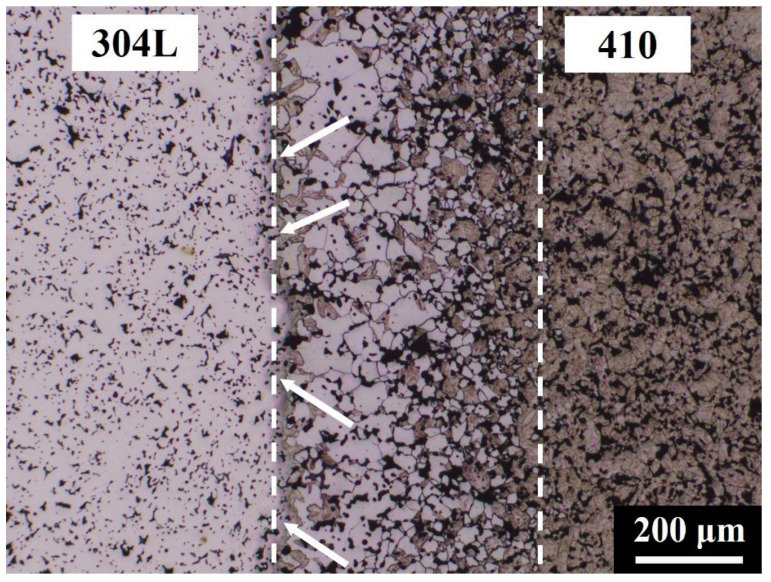
OM microstructure at the interface of the 410/304L LMC.

Figure 5 shows the microstructure at the interface of the 410/304L LMC. The microstructures in the 410 and 304L sides should have been martensite and austenite, respectively. However, the results showed unexpected microstructures, namely, the white and dark phases outlined by dashed lines in Figure 5, at the 410/304L interface. As can be seen in the figure, the fractions of the white and dark phases obviously varied with the distance from the interface. These microstructures may have been caused by the mutual diffusion of elements during sintering. 

**Figure 6:**
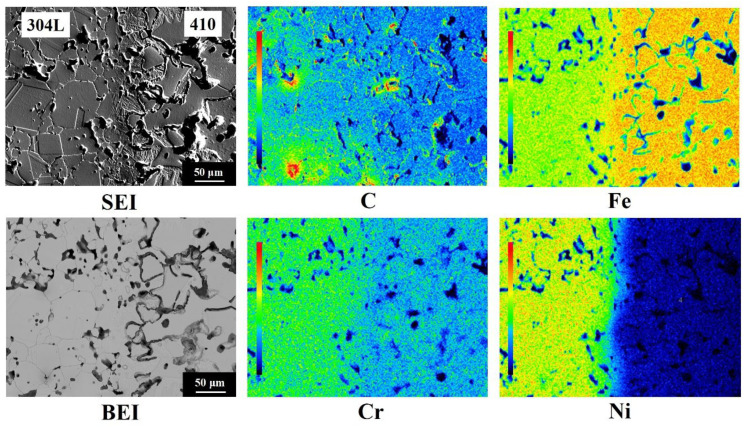
EPMA elemental distributions at the interface of the 410/304L LMCs.

The EPMA elemental distributions at the interface of the 410/304L LMC are shown in Figure 6. The color bar in the left part of each elemental mapping represents the relative levels of atomic concentrations, and the red and the black colors indicate the highest and lowest atomic concentrations, respectively. Because the nominal compositions of the 304L and 410 layers were Fe-18.5Cr-11.3Ni and Fe-12.5Cr-0.4C, respectively, the Cr and Ni contents in the 304L layer should have been higher than those in the 410 layer. Conversely, the Fe and C concentrations in the 410 layer should have been higher than those in the 304L layer. As expected, the EPMA elemental mappings in Figure 6 demonstrate that the 304L layer was rich in Cr and Ni, and the 410 layer was rich in Fe. Before sintering, the C concentration of the 410 layer was higher than that of the 304L layer. Surprisingly, the C mapping in Figure 6 showed that, after sintering, the color levels in the 304L and 410 layers were mostly green and blue, respectively, indicating that the C content of the 304L layer was higher than that of the 410 layer. This phenomenon could be attributed to the difference in the chemical potential of C in the two layers. Wu et al. [17] used thermodynamic simulation to simulate the effects of Cr, Ni, and Mo on the chemical potentials of Fe-X-0.5C at 1120 °C. According to the thermodynamic simulation results, 1 wt % Cr could reduce the chemical potential of C by 1.08 kJ/mol, and 1 wt % nickel could increase the chemical potential of C by 0.44 kJ/mol. Since the Cr and Ni contents of the 304L layer were, respectively, 6 wt % and 11.3 wt % higher than those of the 410 layer, the chemical potential of C was lower in the 304L layer than in the 410 layer. This phenomenon led to the diffusion of C from the 410 layer into the 304L layer during sintering and the formation of a low C area near the interface of the 410 layer.

### 3.4. Phase Identification of Stainless Steel LMC

**Figure 7:**
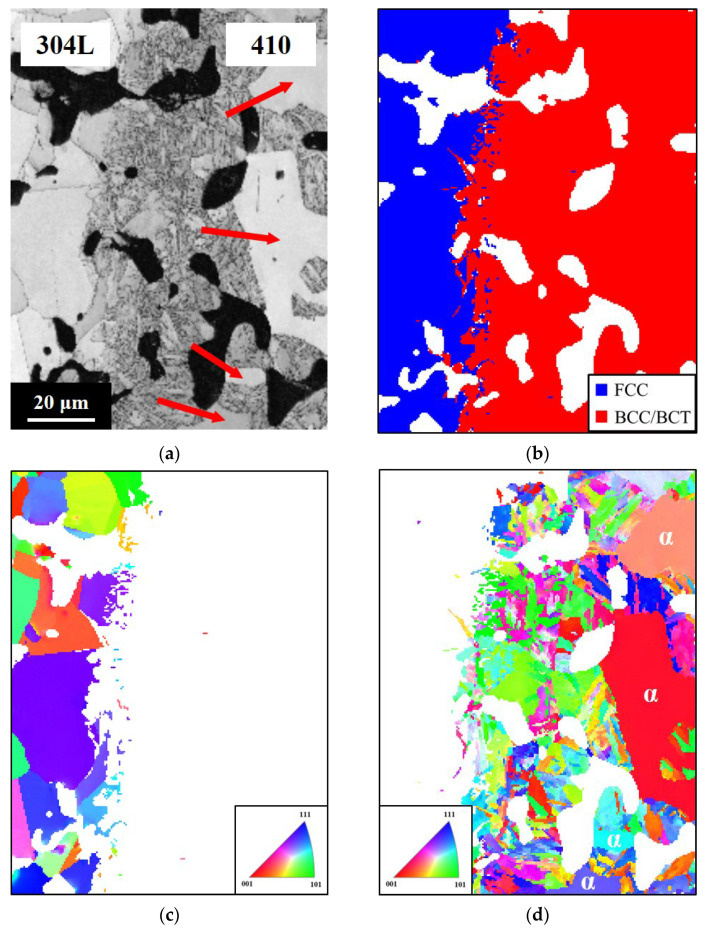

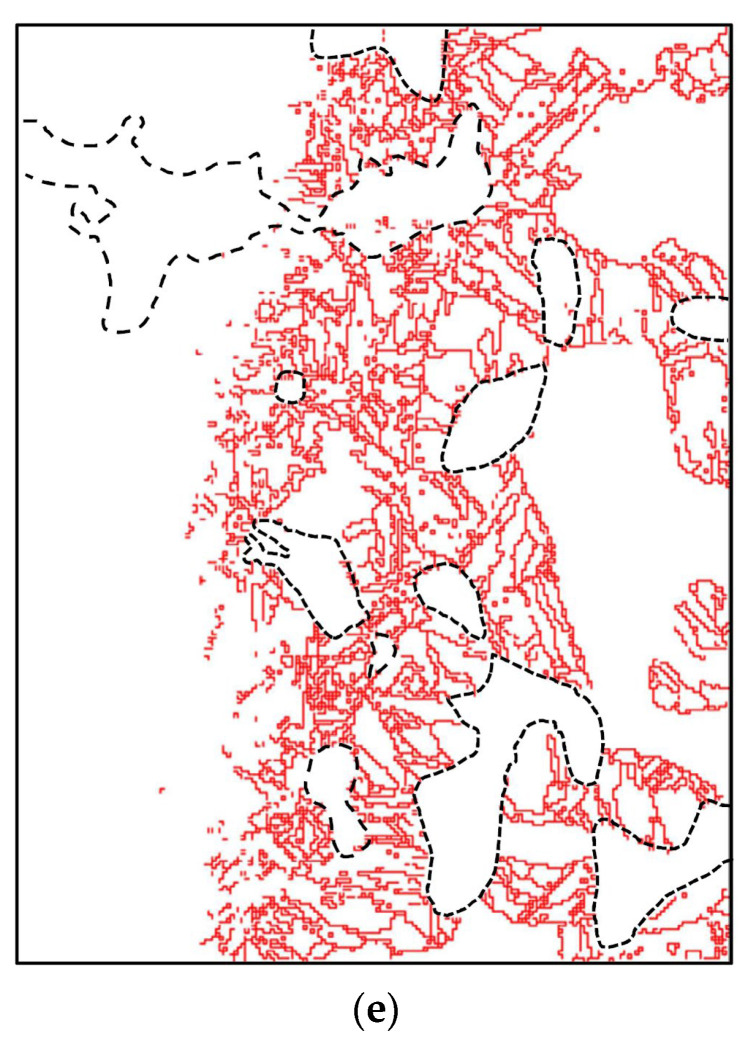
EBSD results of the 410/304L interface. (**a**) Image quality map; (**b**) phase map; (**c**) crystal orientation map of FCC structure; (**d**) crystal orientation map of BCC/BCT structure; (**e**) map of low-angle grain boundary in BCC/BCT structure.

The secondary electron image (SEI) in Figure 6 shows an acicular structure with a thickness of about 50 μm at the 410/304L interface. Moreover, the microstructure of the 410 side was not martensite. The crystal structures near the 410/304L interface were identified by EBSD and are shown in Figure 7. The results indicated that the 304L layer was FCC (austenite), and both the 410 layer and the acicular structure at the 410/304L interface were BCC/BCT structures. The image quality map and crystal orientation map clearly showed that the two regions identified as BCC/BCT exhibited different characteristics. The acicular structure had a lower image quality than the coarse grains in the 410 layer, as indicated by the arrows. The microhardness of the coarse grains with a higher image quality and the acicular structure with a lower image quality were, respectively, 197 HV and 484 HV. The large difference in microhardness between the two areas implied that they could not be the same phase, although both were identified as BCC/BCT in structure. 

The distributions of low-angle grain boundaries (angle < 10°) marked by the solid red lines are displayed in Figure 7e. Some boundaries between the pores and the areas identified as BCC/BCT structure were also marked by the dashed black lines in Figure 7e. The results show that numerous low-angle grain boundaries existed inside the acicular structure with a lower image quality. The image quality map in EBSD reflects the crystal perfection in the analyzed areas [18]. Defects in the crystal structure, such as dislocations or grain boundaries, impair the intensity of Kikuchi patterns and thus degrade the brightness of the image quality map in EBSD. In an Fe-based alloy, martensite contains numerous crystal defects, including dislocations, twin boundaries, and lath boundaries [19]. Because the defect concentration of martensite is obviously higher than that of ferrite, the image quality of the martensite was inferior to that of ferrite [17,20]. Thus, the coarse grains with superior image quality/lower microhardness and the acicular structure with inferior image quality/higher microhardness were ferrite and martensite, respectively.

The generation of ferrite with a thickness of about 200 μm near the interface of the 410 layer, as shown in Figure 5, can be ascribed to the uphill diffusion of C from the 410 layer into the 304L layer. Figure 5 also shows that the fraction of ferrite near the interface of the 410 layer gradually decreased at greater distances from the interface, while the fraction of martensite increased to 100% at greater distances from the interface. Moreover, Figure 7 indicates the presence of a martensite layer with a thickness of about 50 μm at the 410/304L interface. During sintering, Cr and Ni gradually diffused from the 304L layer to the 410 layer, although the diffusion distance was not far. The high Cr and Ni content near the interface of the 410 layer enhanced the hardenability and led to the formation of martensite, even though the C content in this region was low.

Figure 2, Figure 5 and Figure 7 show no cracking or delamination at the interface, indicating that the interfacial bonding was superior. With proper powder filling and pressing, the LMC specimens could be successfully produced without interfacial cracks. Since the particle size and sintering driving force of these raw powders were similar, no interfacial cracks or distortion resulted from the different shrinkage rates of the layers after sintering. This finding demonstrates that the PM process is a feasible technology for fabricating LMCs.

## 4. Conclusions

The PM process with adequate processing parameters is a feasible means of producing LMCs. After sintering, no interfacial cracks or distortion were observed, indicating superior bonding between the layers.

In the Fe-4Ni-3Cr-0.5Mo-0.5C/Fe LMC, a continuous pearlite layer was generated between the Fe-4Ni-3Cr-0.5Mo-0.5C and Fe layers after sintering. Furthermore, diffusion of C from the Fe-4Ni-3Cr-0.5Mo-0.5C layer to the Fe layer led to the formation of a ferrite/pearlite mixture in the Fe layer.

In the 410/304L LMC, the difference in the chemical potentials of C between the 410 and 304L layers resulted in the uphill diffusion of C from the 410 layer to the 304L layer. A continuous ferrite layer thus formed near the interface of the 410 layer. At the interface, a continuous martensite layer was generated due to the high Cr and Ni contents in this region.

## Data Availability

The raw data required to reproduce these results cannot be shared at this time as the data also forms part of an ongoing study.
